# Theoretical models for branch formation in plants

**DOI:** 10.1007/s10265-019-01107-9

**Published:** 2019-04-19

**Authors:** Akiko Nakamasu, Takumi Higaki

**Affiliations:** International Research Organization for Advanced Science and Technology, Kumamoto University, 2-39-1 Kurokami, Chuou-ku, Kumamoto, 860-8555 Japan

**Keywords:** Branch, Divarication, Mathematical model, Plant morphogenesis, Theoretical approach

## Abstract

Various branch architectures are observed in living organisms including plants. Branch formation has traditionally been an area of interest in the field of developmental biology, and theoretical approaches are now commonly used to understand the complex mechanisms involved. In this review article, we provide an overview of theoretical approaches including mathematical models and computer simulations for studying plant branch formation. These approaches cover a wide range of topics. In particular, we focus on the importance of positional information in branch formation, which has been especially revealed by theoretical research in plants including computations of developmental processes.

## Introduction

Branch architectures are observed in various plant organs including shoot meristems (Kuhlemeier 2007, 2017), inflorescence stems (Bommert and Whipple 2018), leaves (Efroni et al. 2010; Wang and Jiao 2018), roots (Hinsinger et al. 2005), prothallia (Momose 1967) and thalli (Parihar 1967). On a cellular level, the morphogenesis of some unicellular algae (Lacalli 1975a, b), leaf pavement cells (Higaki et al. 2017), and root hair cells (Payne and Grierson 2009) can be regarded as branching (Li et al. 2018). The branch formation repeatedly observed in plants is important from a morphological perspective.

Here, we review theoretical models for branch formation in the different hierarchies of plant architecture. These theoretical approaches have been used to understand the complex mechanisms involved. In particular, morphogenesis is a difficult process to image because it involves continuous deformation through localized growth. We can confirm the feasibility of a predicted condition by simulation, therefore simulations using adequate theoretical models have been important for solving the problems in morphogenesis involving branch formation. Theoretical research on animal branch development in recent years has been extensive whereas branch formation in plants has also been investigated traditionally (Cohen 1967; Honda 1971; Lindenmayer 1968, 1971; Meinhardt 1976, 1982). In the present article, we focus on the generation processes, complexity, environmental interactions, dimensionality, and mode selection for branching in plants.

## Triggers for generation of branches

Growth inhomogeneity (i.e., differential growth rates) is the most important factor for generating complexity in plant morphogenesis, although programmed cell death also contributes to the formation of tubular structures, such as aerenchyma and tracheary elements (Jones and Dangle 1996). During organ formations, cell division and subsequent cell expansion show differences in their activities and deployment directions, and thus are reflected in the generation of growth inhomogeneity. Branches are initiated through growth inhomogeneity, and subsequently some branches elongate after their generation.

Mechanical stresses and their feedback via the cytoskeleton are considered to be one factor that controls the growth inhomogeneity in plants (Hamant et al. 2008). Mathematical models including feedback from a numerically predicted pressure distribution in an elastic medium have been applied to shoot meristems (Bozorg et al. 2014) and sepal formation (Hervieux et al. 2016). In this framework, Hong et al. (2016) showed that organs lose their robustness to generate a regular size and shape without spatiotemporal averaging in their growth, i.e., inhomogeneities occur against regular boundaries, although the inhomogeneities are disorganized. Therefore, some mechanisms are needed to arrange the inhomogeneity.

The mechanical aspects of morphogenesis are important considerations for branch formation. For example, the jigsaw puzzle-like shapes of cotyledon pavement cells can be explained by a mechanical model in which excessive cell-wall growth promotes undulation and buckling of lateral cell walls (Higaki et al. 2017). In addition, activation feedback against the convex wall formations can arrange the shape of the jigsaw puzzle-like pattern (Sapala et al. 2018), although the buckling itself can generate furrows with a specific wavelength (Higaki et al. 2017; Takigawa-Imamura et al. 2015). When concave walls are connected by microtubules that prevent expansion, formation of convex wall shapes is activated. This effect subsequently prevents the connection of convex walls. For the initiation of the activation feedback, positional information such as distributions of Rho of plants (ROP) proteins may be related in.

Molecular-based positional information, especially existing as periodic patterns, is important for plant branch formation (Lacalli 1975b; Meinhardt 1982; Meinhardt and Gierer 1974). As suggested by Turing (1952), the mechanisms underlying the formation of such patterns can be explained by reaction–diffusion (RD) systems. The conditions required for RD pattern formation can be applied to arbitrary interactions of molecules such as WUSCHEL–CLAVATA in the shoot meristem (Fujita et al. 2011), and SPEEACHLESS–SCREAM and EPIDERMAL PATTERNING FACTOR 2 in stomatal positioning (Horst et al. 2015). On the cellular level, ROP proteins are considered to be crucial factors that provide the positional information observed in root hairs (Jones et al. 2002; Molendijk et al. 2001), pavement cells (Fu et al. 2005), and tracheary elements (Nagashima et al. 2018). The ROP localization was explained within the framework of a RD model (Nagashima et al. 2018; Payne and Grierson 2009).

In addition, PIN-FORMED (PIN)-mediated polar auxin transport (PAT) is considered to provide various types of positional information in plants. Though many mathematical models have been proposed for the required traits of positional information, the self-organization properties of auxin and different directions of PIN and auxin patterns against auxin flux, that summarized by van Berkel et al. (2013), indicated inadequacies of the models.

## Simple to complex branches

**Fig. 1:**
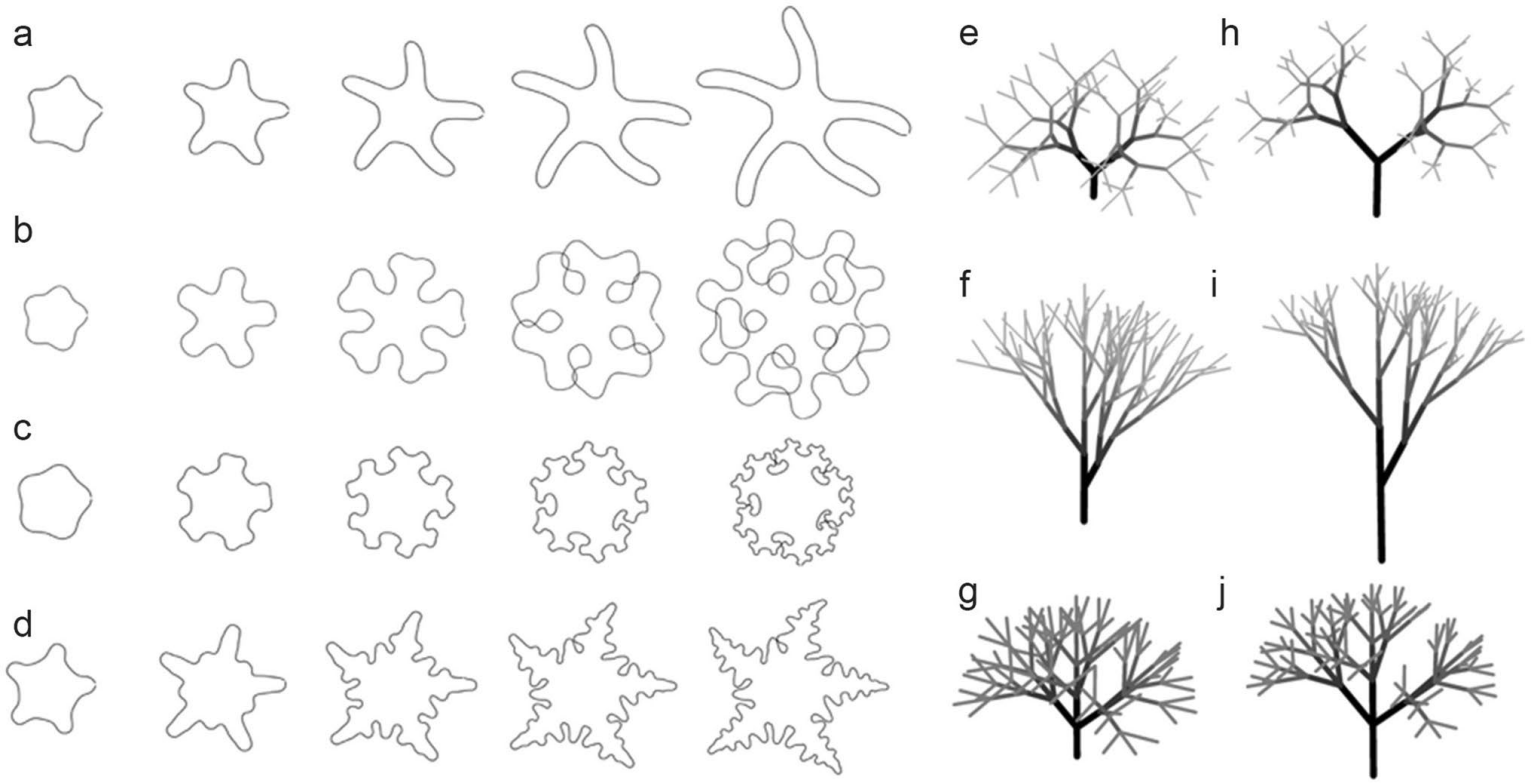
Diversity of branches. **a**–**d** Development of diversity in divarication: from left to right, disk-like architectures grown with equally spaced periodic patterns, as described by Harrison and Kolář (1988), Holloway and Harrison (1999), and Nakamasu et al. (2014). Branches gradually develop during growth processes. **a** No side branches, **b**, **c** bifurcation, and **d** monopodial branching. As branch development proceeds, the branches tend to overlap. **c**, **d** Scale-downs of iteratively added units to avoid collision is included. **e**–**j** Representations of three-dimensional branching with particular branching rules were generated based on Honda’s I-model (Borchert and Honda 1984); **e**, **h** bifurcation; divergent angle is 90° and branch angle of two daughter branches is 45°; **f**, **i** alternate phyllotaxis; divergent angle is 137.5° and branch angle is 45°; and **g**, **j** opposite phyllotaxis; branch angles of two lateral branches are 45° and divergent angle is 90°. **e**–**g** Branch lengths are the same for the whole tree. **h**–**j** Branch lengths decrease dependent on the branch hierarchies with ratio 0.8

To generalize plant branch formation, here we consider situations where disk-like architectures gradually produce branches during their growth processes (Fig. 1a–d). When the apparent branching rules of the branches are different, differences become prominent as growth progresses as shown in Fig. 1. Complex architectures, such as nested branches (i.e., branches hierarchically composed of side branches; Fig. 1b–d, e–j) tend to overlap (Harrison and Kolář 1988; Holloway and Harrison 1999; Nakamasu et al. 2014), e.g., the disc developed in a limited space in Fig. 1b. The complexity of branching depends on the frequency of branch generation (i.e., branching times); therefore, to prevent tangled branches, branch generation or subsequent elongation needs to be restricted as Fig. 1a with no side branch. This restriction has effect to maintain the simplicity of branch shape. Leaf-and-flower generation occurs only in the shoot meristem; therefore, not only spatial restriction but also temporal continuums of shoot meristem identity create different branch architectures from simple to complex and more (Prusinkiewicz et al. 2007). Furthermore, formation of a fractal structure by the scale-down of repeating units enables efficient use of the limited space, thus avoiding branch overlap without interrupting the generation of complexity (Holloway and Harrison 1999) (Fig. 1c, d, h–j). Shortened branch lengths were also incorporated into tree-like architectures in Honda’s model (Honda 1971). As shown in Fig. 1e–j, different branching rules show equivalent results to the contraction of branch length in three-dimensional trees based on Honda’s model (Borchert and Honda 1984; Honda 1971). These localized and/or fractal-like branch architectures have many examples in plants as follows. Restriction of branch generation is observed in situations where the number of inflorescence branches is increased (Nakagawa et al. 2002) or leaves are dissected (Berger et al. 2009; Larue et al. 2009) by genetic manipulations. Self-similarities are observed as fractal structures in leaf veins, canopies (Mandelbrout 1983), fern leaves (Barnsley 1988), and Romanesco cauliflower.

The two-dimensional branch architectures termed divarications are often observed in leaves. Such divaricated leaves are categorized as serrations, lobes, and leaflets mainly according to their degree of protrusion. Simple and compound leaves are sharply distinguished from one another, though the arrangement of lobes or leaflets in divarications often show commonalities (Nakamasu et al. 2017). A heterophyllous plant, *Rorippa aquatica*, has sequential peripheral complexity from an elliptically shaped simple leaf to a finely dissected leaf (Nakayama et al. 2012). This type of heterophylly can be understood by the model that combines both strategies mentioned above (A. Nakamasu, N. J. Suematsu, and S. Kimura, unpublished data). In the model, spatial restrictions explain the formation of simpler shapes, as shown in leaves in *Arabidopsis thaliana* L. (Bilsborough et al. 2011). Also, the relative reductions of spatial scale permit the natural tapering of divarication units toward the distal ends and avoid overlaps, as reported in Holloway and Harrison (1999).

## Open or closed branches

**Fig. 2:**
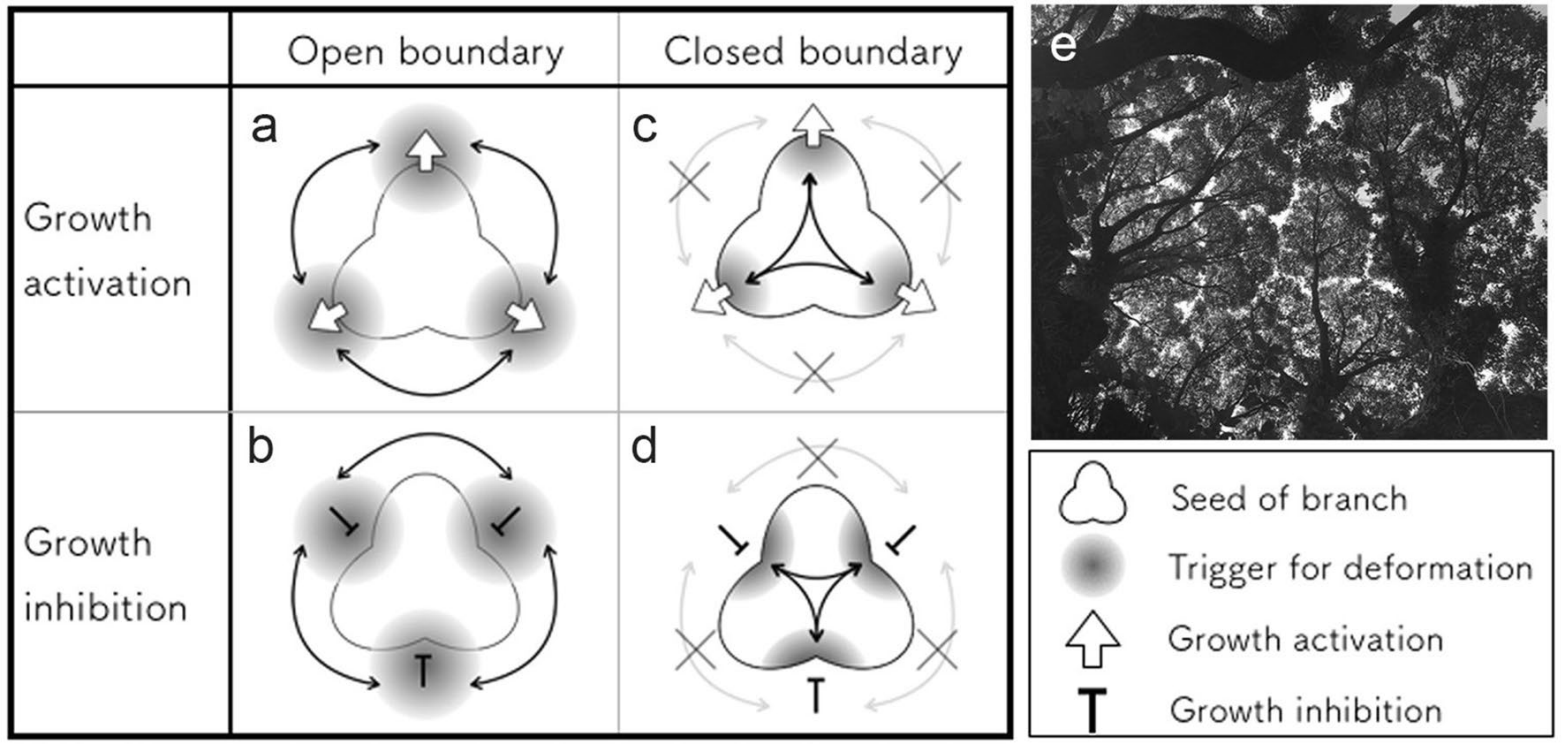
Interactions with the external environment in branch formation. The formation of branch architecture in plants is considered to be produced by changes in growth intensity, which seem to include activation (**a**, **c**) and inhibition (**b**, **d**) of growth. Each case includes open boundaries (**a**, **b**) and closed boundaries (**c**, **d**). With open boundaries, branches can interact via the external environment (**a**, **b**). By contrast, with closed boundaries, the generated branches need to avoid overlaps (**c**, **d**). In some cases, even branches with closed boundaries can be modified by the local environment. **e** A crown shyness-like phenomenon observed in camphor trees, which is considered to be an example of the local interaction

In general, the branches of the aerial parts of plants, such as the body plan and leaf shape, have abundant variety and reproducible characteristics, and these branches are often used for taxonomic identification. Conversely, the underground branches of roots have less consistent features and tend to show phenotypic plasticity depending on their chemical and physical environments (reviewed in Hinsinger et al. 2005). The importance of environmental interactions in branching was recognized in early period on theoretical researches and its effects have been implemented into simulation of branch formation (Cohen 1967; Honda and Hatta 2004; Honda et al. 1997). Mech and Prusinkiewicz (1996) summarize the incorporation of such interactions into models such as L-system (Mech and Prusinkiewicz 1996). In this section, we focus on the boundaries between branches and the environment, and whether they are open or closed. With open boundaries, branches can interact via the external environment, but in the case of a closed boundary, branches can only recognize others (if possible) internally (Fig. 2).

Plant branches elongate in a space-filling manner, resulting in the generation of evenly distributed shapes. In cases of stochastic formation, the generated branches tend to disrupt the whole regularity. To avoid branch collision in this case, mutual interactions between the flexible environment and branch generation are needed, such as diffusion-limited aggregation (Witten and Sander 1981). A similar situation was also postulated in the phase field model used to express crystal growth (Kobayashi 1993). These branches are considered to need an open boundary to reduce branch overlap. As one such example in plants, leaf veins can be considered to be a branch architecture having an open boundary with the blade as an external environment (Fig. 2a, b). Although leaf veins also follow the same rule for prevention of overlaps, loops (i.e., the collision of branches) are permissible in some exceptions. In mathematical models for leaf vein formation, the boundary between a blade and a premature vein is open, and molecules that determine the vein position can diffuse through the boundary whether flows are involved (Fujita and Mochizuki 2006; Mitchison 1980, 1981) or not (Meinhardt 1976). Such physical gradients spreading at the multicellular scale can react and have an effect within this range. This enables evenly distributed vein positioning with several cell intervals. Root formation can also be considered to have an open boundary. Both soil conditions and several types of feedback from the absorption of substrates by the root itself regulate rhizosphere geometries (Hinsinger et al. 2005; Walter et al. 2009). Historically, morphological analyses of roots architecture have been difficult, but recent technical advances should allow theoretical studies to progress (Band et al. 2012; Keyes et al. 2017).

By contrast, most types of branch formation in plants are considered to involve deformation with a closed boundary (Fig. 2c, d). It is well known that various types of intrinsic regulation of branch angle can produce the branch patterns observed in reality (Fisher and Honda 1977; Honda 1971) (Fig. 1e–j). It has been also proposed that appropriate rules for branch formation can reproduce the various traits of plants from a series of studies using L-systems (Prusinkiewicz and Lindenmayer 1990). Restriction of the mutual interactions between branches through the external environment is expected to results in branches lose their positioning control easily. However, spiral phyllotaxis tends to show divergence close to the golden angle, which is advantageous for avoiding overlaps when seen from above (Niklas 1988). So, how are such rules derived? It was pointed out that positional information with spatial periodicity is important for the positioning of the repetitive units (Meinhardt 1982). Positional information with a specific wavelength can be explained by molecular interactions within a boundary (Jonsson et al. 2006; Smith et al. 2006; Turing 1952).

Leaf primordia are formed in the “first available space” (Adler 1974; van Iterson 1907). From a geometrical perspective, it was suggested that equal spaces between primordia are essential to form the golden angle (Mitchison 1977; Richter and Schranner 1978). Phyllotaxis patterns including the golden angle arrangement can be reproduced by simulations of iterative insertion of a new primordium in the most distant place from the previous leaf primordia on a two-dimensional curved surface (Jonsson et al. 2006; Smith et al. 2006). Though some researchers suggested that temporal regulation or mechanical force are related to the patterning (Douady and Couder 1996a, b; Shipman and Newell 2004), these discussions and historical backgrounds are excellently summarized in Kuhlemeier 2007, 2017.

**Fig. 3:**
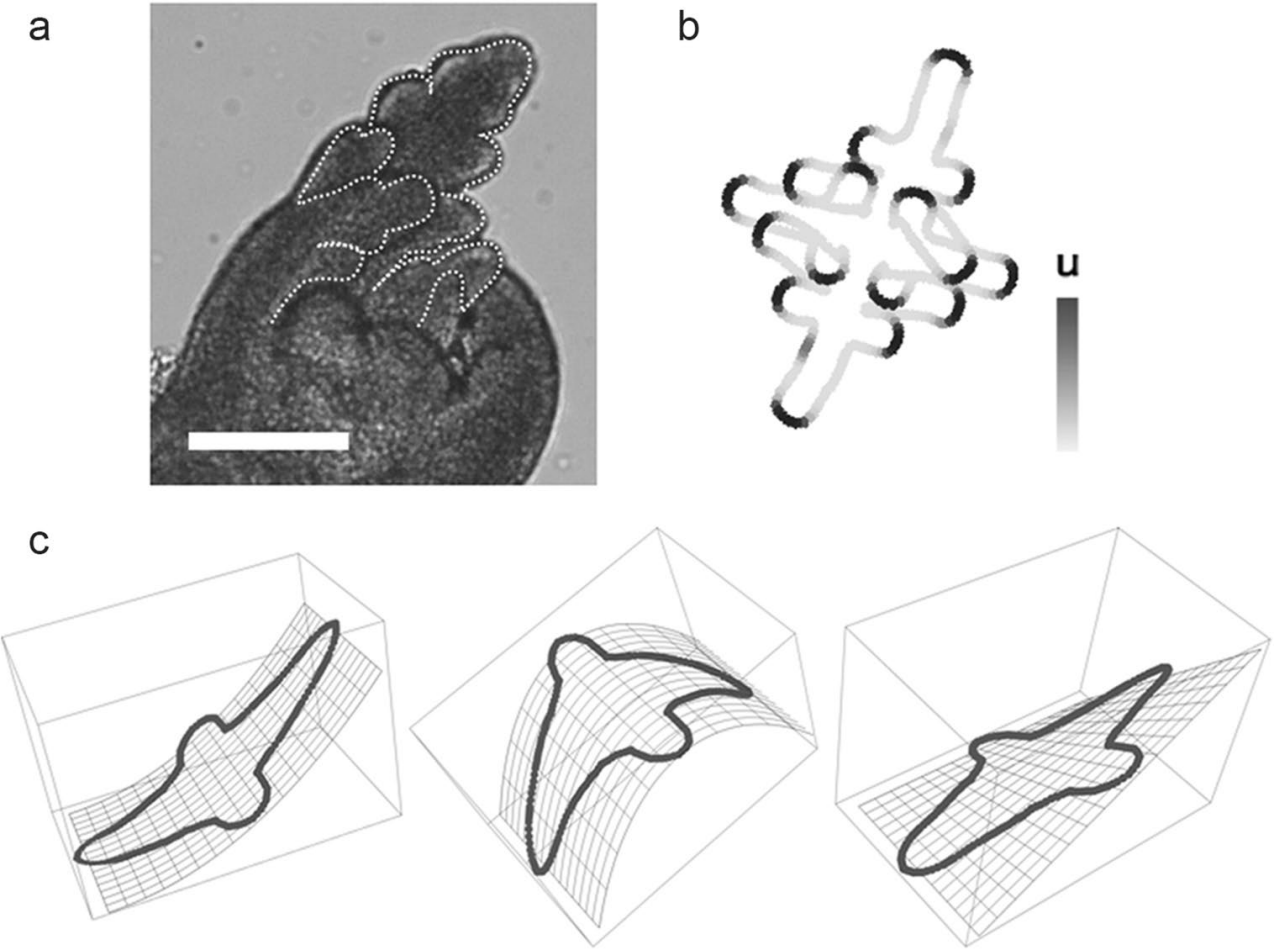
Deformations of circumference in three-dimensional spaces. **a** A leaf primordium in a shoot apical meristem of *Eschscholzia californica* Cham.; scale bar 100 µm. **b** Divarication generated by deformation of a ring on a two-dimensional plane. The ring was grown based on an equally spaced periodic pattern (based on the model in Nakamasu et al. 2014). **c** Deformations of divarication on a two-dimensional plane in a three-dimensional space. **a** The boundary between the adaxial and abaxial sides of the primordium is outlined with a white dashed line. Continuous deformation of the boundary is considered to correspond to the deformation of a ring on a two-dimensional plane

Periodic growth of a boundary in a two-dimensional plane is another example of branch formation with a closed boundary. Various divarications can be generated by models using different traits with periodicity (Harrison and Kolář 1988; Holloway and Harrison 1999; Nakamasu et al. 2014). Contributions other than from the boundary are not considered for such branch positioning, and reproducible steady-state shapes can be obtained. A regular sequence of branch arrangements is observed when a ring is just grown depending on an equally spaced periodic pattern. Such programmed divarications were modeled first with bifurcation and subsequently with lateral branching. (Harrison and Kolář 1988; Nakamasu et al. 2014) (Figs. 1b–d, 3b). In the case of lateral branching, the sequence of regular arrangements observed in the intact (i.e., not modified) branches was described using recurrence formulas (Nakamasu et al. 2014, 2017), and is coincidentally comparable to a specific parameter in (tD)OL-systems with a delay (Prusinkiewicz and Lindenmayer 1990), though these models are completely different systems. The former utilizes the spatial scale to make form, whereas the latter model repeatedly adds a stable unit as measured in time to adjust the branch arrangement. This rule is appropriate to explain branch arrangements that include asymmetry observed in actual leaves (Nakamasu et al. 2014, 2017). Degree of branch asymmetry can be expressed as subtractions of sequential steps of the recurrence formula. Continuous growth based on intrinsic periodicity can generate a certain branch pattern also in three dimensions, as exemplified by Holloway and Harrison (2008).

Regardless of whether the boundary is open or closed, branch generation is often modified by interactions with a comparatively neighboring (i.e., local in Mech and Prusinkiewicz 1996) environment. In addition to responsive roots, deterministic branches with a closed boundary can also avoid obstacles. For example, “crown shyness”-like phenomena in which tree crowns compete or yield space to each other are considered to be a good example of the local modification of plant branching (Fig. 2e). As an additional example, it was reported that leaf shapes are in good agreement with Kirigami (folded-cut paper; Couturie et al. 2011), which suggests that mechanical forces are involved in leaf organogenesis. Leaf primordia packed in a bud have restricted space then folded, therefore, mechanical forces generated by physical contacts regulate leaf growth, resulting in geometrical constraints of peripheral expansion. As exemplified in this section, mechanical forces are associated with such modifications.

## Two or three dimensions for branches

Branching does not occur in one dimension, it requires two or more dimensions. When we focus on a leaf indentation, a boundary can be captured as a one-dimensional curved line on a two-dimensional plane (Bilsborough et al. 2011; Nakamasu et al. 2014; Prusinkiewicz and Runions 2012) (Fig. 3). Though leaf primordia grow three-dimensionally as shown in Fig. 3a, almost all leaves can be considered to be two-dimensional planes with the adaxial and abaxial sides already specified (McConnell et al. 2011; Sawa et al. 1999; Siegfried et al. 1999). Therefore, the boundary is assumed to be equivalent to a rubber band (i.e., a one-dimensional circumference) in three-dimensional space (Fig. 3a). In many cases, deformations of the rubber band in three-dimensional space can be described similarly to that on a two-dimensional plane (Fig. 3c). That is, the simulated divarication on two-dimensional plane can exist in three-dimensions. The two-dimensional implementations are effective and efficient even if there are overlaps, because the three-dimensional space is practically useful to avoid the inevitable collision of divarication in two dimensions (Fig. 3a, b).

By contrast, because most plant organs develop from meristems (two-dimensional curved surfaces), branch positions need to be understood three-dimensionally (Honda 1971). In general, higher dimensions increase the degrees of freedom but are not simply applicable to the complexities of actual plant branches, such as when imaging young shoots of edible asparagus that retains a simple shape. A two-dimensional branch in three dimensions has more dynamic degrees of freedom than a three-dimensional branch in three dimensions. That is, the branch can escape in the vertical directions from leaf plane. Therefore, overlap on a two-dimensional plane is less critical. However, a three-dimensional branch cannot escape to four dimensions, so overlaps in three dimensions are unacceptable. Three-dimensional branches desperately need to avoid collision. Therefore, in plants that continue to form branch architectures throughout their lifetime, branching should be frequently confined to a limited region.

**Fig. 4:**
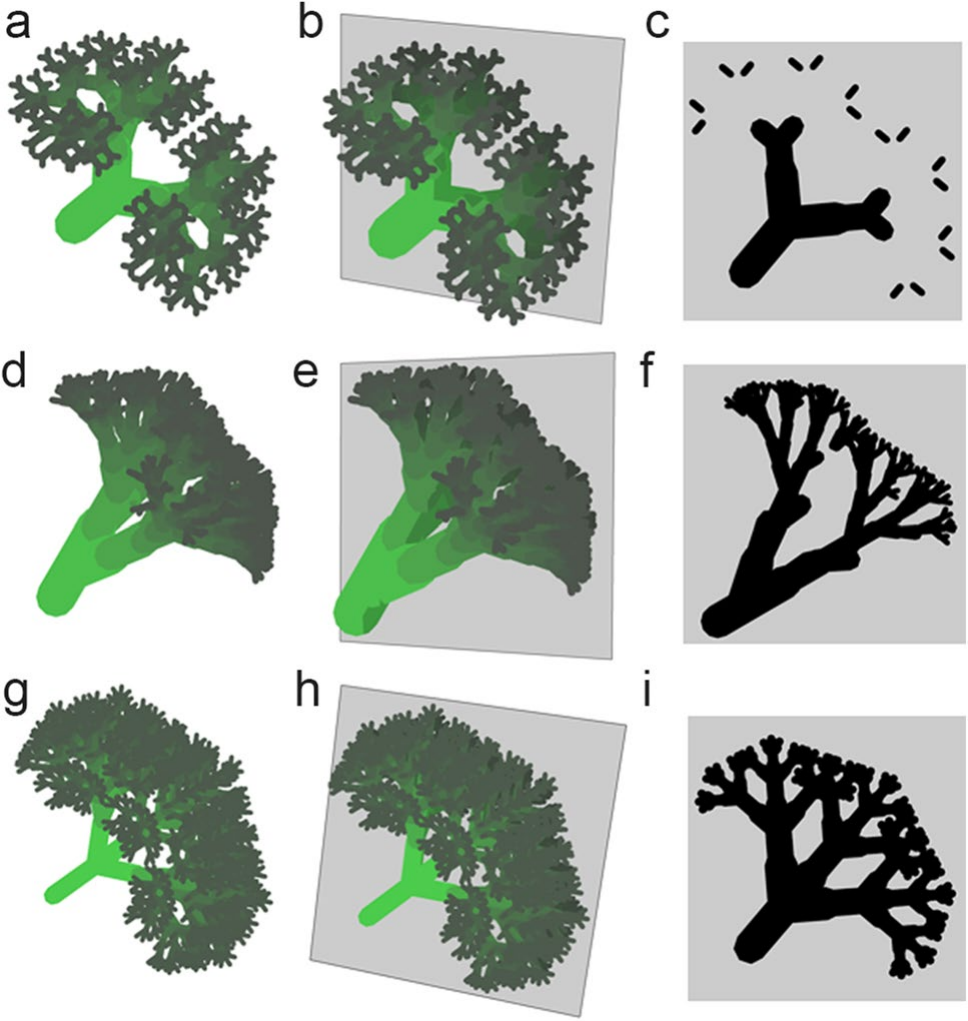
Longitudinal sections of virtual broccoli inflorescences. Each row shows a virtual broccoli inflorescences and its section generated by the same branch rules shown in Fig. 1h–j. **a**–**c** Bifurcation, **d**–**f** alternate phyllotaxis and **g**–**i** opposite phyllotaxis with lateral branching based on Honda’s I-model (Borchert and Honda 1984). **d**–**f** The middle row shows a similar model to actual broccoli (the divergence angle is 137.5°). **b**, **e**, **h** Each broccoli inflorescences is dissected through the gray plane. **c**, **f**, **i** The expected cross-sections of each broccoli inflorescences

In Fig. 4, virtual broccoli inflorescences with different branch rules were generated and then longitudinally sectioned. The rules used for each row of imagings in Fig. 4 correspond to those in Fig. 1h–j. Compared with Fig. 3b, two-dimensionally formed divarications are not simple longitudinal-sections of three-dimensional branches (Fig. 4c, f, i). Therefore, different discussion frameworks should be applied to two- and three-dimensional branches.

## Different modes of branches

**Fig. 5:**
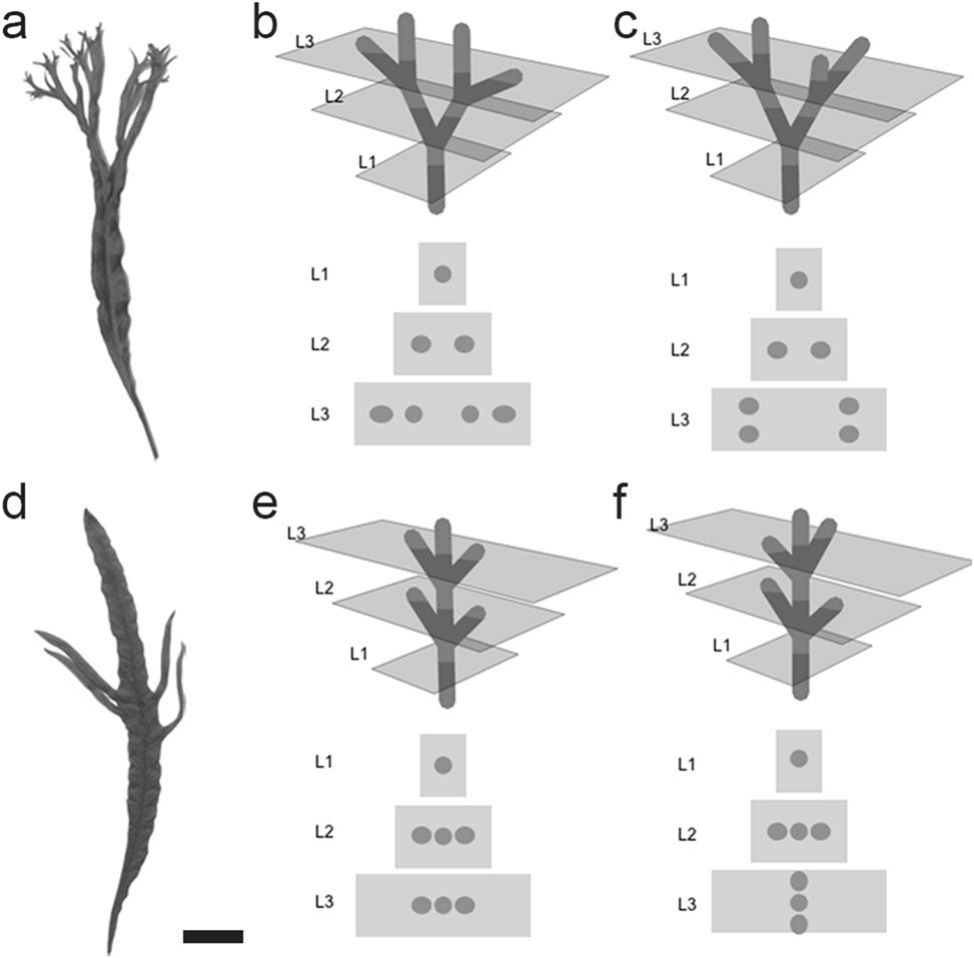
Different modes of branching. Contrasting modes of divarication observed in leaf shapes in closely related ferns; **a** bifurcated *Microsorum pteropus* Copel. var. *windelov* and **d** laterally branched *Microsorum* sp. ‘Fork leaf’, based on Miyoshi et al. (2019). Scale bar 2 cm. **b**, **c**, **e**, **f** Three-dimensional diversity in each mode. **b**, **c** Bifurcation and **e**, **f** lateral branching. Each branch was sectioned horizontally in the gray plane. The sections of each layer (L1–L3) is shown beneath each branch

Branches often show two different structural modes, which are classified as bifurcation and lateral branching, as exemplified in Figs. 1, 4, and 5. The mode differences are observed in both two- or three-dimensional branches in leaves (Miyoshi et al. 2019) or body plans (Harrison 2017) (Fig. 5). However, three-dimensional branches are more diverse within a certain branch mode (Fig. 5b, c, e, f). The different modes can coexist even in the same organ of a single plant. For example, a liverwort gametophyte shows different modes of branching (Inoue et al. 2008). That is, a system that can select coexisting or independent modes is important for the formation of such branches. In leaves, the two different modes can be observed in closely related fern species (Miyoshi et al. 2019) (Fig. 5a, d). In addition, the formation of bifurcated leaves can be observed in *pin* mutants of *A. thaliana* (Reinhardt et al. 2003). Mutations of *slm1* (a PIN homolog) result in phyllotaxis and leaves showing the bifurcation mode in *Medicago polymorpha* L. (Zhou et al. 2011). It is known that the both modes can be described by patterning—growth coupling (Harrison and Kolář 1988; Holloway and Harrison 2008; Nakamasu et al. 2014). Then, the difference between the branch modes is considered to be explained by the difference in frequency-doubling of pattern, which can be treated as problems of pattern transition on a growing surface (Fujita et al. 2011; Holloway and Harrison 2008). PIN-mediated polar auxin transport is known to be a mechanism that can generate periodic positional information (Bilsborough et al. 2011; Jonsson et al. 2006; Smith et al. 2006; van Berkel et al. 2013). It is interesting that a different branch mode (bifurcation) becomes apparent with the loss of PIN function (Harrison 2017).

## Conclusion

In this review, we first considered the mechanical aspects of branch formation and pattern-formation mechanisms by plant molecules. Second, we reviewed the effectiveness of spatiotemporal restriction of branch generation and the scale-down of added units to avoid the overlap that accompanies increasing branch complexity. We then discussed interactions with the external environment during branch formation. In addition, we presented examples of the differences between two- and three-dimensional branches, and the variation in branching modes deployed in these dimensions.

Recently, understanding of divarication based on equally spaced periodicity has progressed. The mechanism (i.e., patterning–growth coupling) ensures the generation of reproducible and regular divarication arrangements not only in bifurcation but also in monopodial branching. It is considered to be a two-dimensional version of programmed branching observed in the development of murine lungs by Metzger et al. (2008). Divarication is characteristic of plant leaves, therefore this finding is an achievement of plant derivation. However, actual leaves frequently show branch patterns different from the sequence of regular arrangements. Therefore, we need to consider such differences from the perspective of modification of the deterministic rule. Three-dimensional branching based on periodic positional information remains unexplored, though some reproducible branch arrangements have been predicted from the golden angle of divergence in phyllotaxis and the branch pattern of early lung formation in mice (Metzger et al. 2008). Frameworks to deal with deformation in three-dimensional space are needed for such studies, and related equipment has been developed (Matsuda et al. 2017; Okuda et al. 2018) and partially investigated. Plants with closed boundaries in which the intrinsic branching patterns tend to be maintained may be advantageous for this kind of investigation. Because almost all branched organ formations in animals are considered to include interactions with the surrounding environment (Iber and Menshykau 2015; Miura 2015), the resulting branches might be somewhat different from the intact arrangements. The differences in branching modes that are commonly observed in plants and animals are parallel problems, and have been treated theoretically in limb, lung and kidney formation in animals (Hirashima et al. 2009; Menshykau et al. 2012; Miura et al. 2006; Xu et al. 2017). Then the factors which determined the mechanism of transition were mathematically analyzed (Crampin et al. 2002). To address this problem, an understanding of positional information at the molecular level is required. Subsequently, the plant-specific pattern-formation mechanism of the PAT system, which is described by many models, should be integrated. On these points, references from case studies of animal may be relevant for future perspectives.
