# Correction to: Theoretical models for branch formation in plants

**DOI:** 10.1007/s10265-020-01178-z

**Published:** 2020-03-25

**Authors:** Akiko Nakamasu, Takumi Higaki

**Affiliations:** International Research Organization for Advanced Science and Technology, Kumamoto University, 2-39-1 Kurokami, Chuou-ku, Kumamoto, 860-8555 Japan

## Correction to: Journal of Plant Research (2019) 132:325–333 https://doi.org/10.1007/s10265-019-01107-9

The article Theoretical models for branch formation in plants, written by Akiko Nakamasu and Takumi Higaki, was originally published Online First without Open Access. After publication in volume 132, issue 3, pages 325–333 the author decided to opt for Open Choice and to make the article an Open Access publication. Therefore, the copyright of the article has been changed to © The Author(s) 2020 and the article is forthwith distributed under the terms of the Creative Commons Attribution 4.0 International License (https://creativecommons.org/licenses/by/4.0/), which permits use, duplication, adaptation, distribution and reproduction in any medium or format, as long as you give appropriate credit to the original author(s) and the source, provide a link to the Creative Commons license, and indicate if changes were made.

The original article was updated.

